# Alterations of spontaneous brain activity in type 2 diabetes mellitus without mild cognitive impairment: a resting-state functional magnetic resonance study

**DOI:** 10.3389/fnhum.2023.1305571

**Published:** 2024-01-11

**Authors:** Qianru Wang, Chuanke Hou, Xingyuan Jiang, Hongjun Li

**Affiliations:** Department of Radiology, Beijing YouAn Hospital, Capital Medical University, Beijing, China

**Keywords:** type 2 diabetes mellitus, cognitive impairment, resting-state, functional magnetic brain imaging (fMRI), spontaneous brain activity

## Abstract

**Background:**

Type 2 diabetes mellitus (T2DM) has been demonstrated an increased risk factor of cognitive impairment or even dementia. Kinds of resting-state functional magnetic resonance imaging indices have been proposed and used to investigate the brain mechanism underlying diabetic cognitive impairment. This study aimed to explore the early changes in spontaneous neural activity among T2DM patients without cognitive impairment by means of multiple rs-fMRI indices.

**Methods:**

T2DM patients without cognitive impairment and age-, sex-, and education matched control subjects were included in this study. Three rs-fMRI indices, namely amplitude of low-frequency fluctuation (ALFF), regional homogeneity (ReHo) and voxel-mirrored homotopic connectivity (VMHC) were computed after image pre-processing. The Montreal Cognitive Assessment (MoCA) was performed to distinguish normal cognition. Brain volume was also evaluated. Correlation analyses were conducted to explore any relationship among rs-fMRI indices and clinical characteristics.

**Results:**

The T2DM patients were detected significantly decreased neural activity in right angular and left prefrontal gyrus including middle and superior frontal gyrus. Increased activities were also observed in left caudate and the supplementary motor area. No correlation between rs-fMRI indices and clinical characteristics was survived after multiple comparison correction. But we observed a significant, but decreased correlation between ALFF and ReHo values in the reported brain areas.

**Conclusion:**

The combination of ALFF, ReHo and VMHC analyses demonstrated abnormal spontaneous neural activity in brain regions which were reported in T2DM patients without cognitive impairment. These results may enhance our understanding of the diabetic brain changes at the early stage.

## Introduction

Type 2 Diabetes mellitus (T2DM) has been accepted as a higher risk of cognitive dysfunction even dementia (Gomez-Guijarro et al., 2023). While little evidence of long-term declines in cognitive function was found in patients group with type 1 diabetes mellitus, people with T2DM are experiencing a greater chance of accelerated cognition decline as a long term effect (Jacobson et al., 2007). In light of the accelerating incidences of T2DM in the world, establishing the impact of T2DM on the brain is important to understand the underlying brain mechanism involved in the etiology of dementia (Sutherland et al., 2017; Beeri and Bendlin, 2020).

Over the past decade, a few studies have tried to pinpoint T2DM related brain changes using magnetic resonance imaging (MRI). Moran et al. (2013) observed regional brain atrophy associated with T2DM and noted that similar distribution of cortical atrophy had been seen in preclinical Alzheimer’s disease. Together with similar studies, lower gray, white or hippocampal volumes may play a key role in the T2DM related cognitive impairment (Espeland et al., 2013; García-Casares et al., 2014a,b; Feng et al., 2021). However, the magnitude of diabetes-associated changes in brain volumes evolves only slowly over time and so does the cognition. Such variation may become detectable several years prior to diagnosis (de Bresser et al., 2010; Biessels, 2013). Since the pathological changes that underlie atrophy do not necessarily reflect neuronal loss, more sensitive markers linked to impaired cognition in T2DM need to be provided to investigate the early effects of diabetes on the brain (Belfort-Deaguiar et al., 2014).

Nowadays, resting-state functional magnetic resonance imaging (rs-fMRI) becomes a frequently used approach to detect early changes of brain function related to T2DM. Musen et al. (2012) measured the brain functional connectivity (FC) in T2DM patients with no structural brain abnormalities or cognitive impairment. The authors demonstrated that reduced brain connectivity preceded the development of atrophy or cognitive impairment. Subsequently, a battery of studies utilized the FC method to track the T2DM related brain dysfunction (Chen et al., 2014; Liu et al., 2016, 2019; Wang J. et al., 2022; Wang M. et al., 2022). However, the FC method needs a prior settlement of seed region, limiting the whole-brain analysis of brain function. Therefore, voxel-wise rs-fMRI indices, manly including amplitude of low-frequency fluctuation (ALFF), regional homogeneity (ReHo) and voxel-mirrored homotopic connectivity (VMHC), have attracted significant research interest in the neural abnormalities of T2DM patients (Cui et al., 2014, 2022b; Zhou et al., 2014; Peng et al., 2016; Wang et al., 2017, 2019, 2020; Shi et al., 2020; Zhang et al., 2020; Wan et al., 2021; Liu M. et al., 2022). Each of these metrics partly contributed to elucidating the functional abnormalities related diabetes, respectively. Therefore, an exploratory study to indicate the intrinsic brain activity by combing various methods may provide more information about the pathophysiological framework in the diabetic brain (Cui et al., 2014; Zhang et al., 2020).

In this study, we applied ALFF, ReHo and VMHC method simultaneously to explore the global spontaneous neural activity in T2DM patients without cognitive impairment. Inspired by former studies, we were especially interested in the stage that before the incidence of cognitive impairment and tried to picture the early changes in diabetic brains. Correlations among rs-fMRI metrics, clinical characteristics and cognition were also performed. This study may help to further understand the neuromechanism of diabetic cognition impairment.

## Materials and methods

### Participants

This study was conducted from March 2020 to February 2022. Patients were recruited from the practices of collaborating endocrinologists in YouAn Hospital, Capital Medical University. The diagnosis of T2DM was based on the latest criteria outlined by the American Diabetes Association (American Diabetes Association, 2018). Patients aged between 45 and 70 years and possessed >6 years of education were qualified for this study. Other detailed requirements for enrolled patients included: disease duration >1 year; regularly treated with hypoglycemic drugs; no history of hypoglycemic episodes; Montreal Cognitive Assessment (MoCA) score ≥26. Control subjects of normal cognitive function (MoCA score ≥26) were recruited in local community and matched to T2DM patients in terms of age, sex and education. The exclusion criteria for all participants included: a history of alcoholism or drug abuse; history of psychiatric disorders; history of brain lesion; hearing/visual difficulties; a rating score of white matter (WM) change >1; contraindications for MRI scan; body mass index (BMI) >25.

This study was reviewed and approved by the local ethic committee. Written informed consent was obtained from all participants.

### MRI data acquisition

All MRI images were acquired via a Siemens 3.0T Trio scanner (Erlangen, Germany) with a 8-channel head coil. Both T2DM patients and normal controls were instructed to keep eyes closed during the scan. The structural 3D T1-weighted (T1w) sagittal images were obtained using a magnetization prepared rapid gradient echo (MPRAGE) sequence: 160 slices; repetition time, 1900 ms; echo time, 2.52 ms; inversion time, 900 ms; voxel size, 1 mm × 1 mm × 1 mm; flip angle, 9°; field of view, 250 mm × 250 mm; resolution, 256 × 256. Functional images were collected using a gradient echo planar sequence: 33 slices; repetition time, 2000 ms; echo time, 30 ms; slice thickness, 4 mm; flip angle, 90°; field of view, 240 mm × 240 mm; resolution, 64 × 64. Fluid-attenuated inversion images were also collected to examine broad WM hyperintensity or lacunar infarcts. Detailed parameters included: 20 slices; repetition time, 8500 ms; echo time, 100 ms; inversion time, 2850 ms; slice thickness, 5 mm; field of view, 250 mm × 250 mm; resolution, 256 × 256.

Voxel-based morphometry (VBM) analysis was performed to identify any brain atrophy attributable to T2DM using Statistical Parametric Mapping (SPM12) toolbox.^1^ The 3D T1w images were segmented into gray matter (GM), WM and cerebrospinal fluid. The segmented images were registered into Montreal Neurological Institute (MNI) template using DARTEL method and further smoothed before statistical map between patient and control groups was generated. Volumes of brain parenchyma were calculated.

### Biometric and cognitive test data

Medical history and clinical data of all participants were collected from medical records and questionnaires. The measurement of blood pressure was repeated at three different time points during the interview and the averaged values were taken. Hypertension was defined as systolic blood pressure (SBP) >160 mmHg, or diastolic blood pressure (DBP) >95 mmHg, or self-reported routine usage of hypotensive agents. Plasma glucose and glycosylated hemoglobin (HbAlc) were measured at fasting, along with the serum total cholesterol, triglyceride, high-density lipoproteins (HDL) and low-density lipoproteins (LDL) levels.

General cognition of all participants was assessed by means of the MoCA scale. MoCA tests were completed before MRI scan. The MoCA test is a friendly tool to assess cognitive abilities, including: orientation, short-term memory, executive function, language abilities, animal naming, clock-drawing test and attention. Compared with Mini-Mental State Exam (MMSE), the MoCA test is better at distinguishing between normal cognition and MCI, while MMSE test is more suitable for monitoring the decline in cognition. This test was performed in a quiet room by a trained research assistant following standard procedure.

### Pre-processing of rs-fMRI data

The Data Processing and Analysis for Brain Imaging (DPABI) toolbox was used with running SPM12 software (Yan et al., 2016). The first 10 of the 200 volumes were discarded because of being considered unstable, leaving 190 volumes. Further pre-processing procedures included: (1) slice timing correction, (2) head motion correction, (3) co-registration between T1w image and fMRI within each subject, (4) nuisance covariates regression (Friston 24 motion, global mean signal, WM signal and CSF signal), (5) non-linear transformation from individual functional space to MNI152 space (3 mm × 3 mm × 3 mm resolution), (6) spatial smoothing with a 6 mm full-width at half-maximum isotropic Gaussian kernel, (7) removing linear trends, and (8) bandpass filtering (0.01–0.1 Hz). Any subjects having head motion of >2 mm translation or >2° rotation will be excluded.

### Rs-fMRI indices for regional characteristics and functional synchrony

Amplitude of low-frequency fluctuation is the mean amplitudes within a specified frequency range (0.01–0.08 Hz in this study). After removal of linear trends and spatial smoothing, the time series of each voxel was filtered and transformed into frequency domain. The square root of the resultant power spectrum was calculated at each frequency and averaged as the raw ALFF value for each voxel. ReHo analysis was performed on the pre-processed images without smoothing operation. This involved performing linear trend and bandpass filtering, generating ReHo maps by assessing the agreement of the Kendall coefficient for the time series of a voxel and its 26 closest neighbors. The individual ALFF/ReHo value of each voxel was transformed to Z score for normalization.

Voxel-mirrored homotopic connectivity method assumes symmetric morphology between hemispheres. Apparently this assumption doesn’t hold for real brains. The functional images should be transformed before VMHC calculation. T1w images normalized to MNI space of all subjects were averaged to generate a mean T1w image. Then a group-specific symmetrical template was created by averaging the left-right version of the mean T1w image. Functional images were transformed to fit the symmetrical template by refined non-linear registration between individual T1w image and the symmetrical template. The voxel-wise homotopic functional connectivity was computed as the Pearson’s correlation coefficient of time series between each voxel and its symmetrical inter-hemispheric counterpart. The VMHC values were also normalized to Z score format.

### Statistical analysis

Demographic and clinical characteristics, as well as cognitive performance, were compared between patients and using SPSS software (version 22; SPSS, Chicago, Illinois, America). Independent *t*-test was used for continuous variables, while χ^2^ test was used for categorical variables. Mann-Whitney *U*-test was used for variables if the variable failed to be consistent with normal distribution. *P* < 0.05 was considered statistically significant.

The inter-group differences of ALFF, ReHo and VMHC values were investigated with the DPABI software. Two-sample *t*-test was performed and the results were determined via multiple comparison correction using Gaussian Random Field (GRF) method. The voxel-wise threshold was set at *P* < 0.001 and the cluster-wise threshold was set at *P* < 0.05.

### Correlation analysis

Associations among ALFF/ReHo/VMHC in specific abnormal brain regions were investigated by correlation analyses between two of these three indices. The mean values of ALFF, ReHo and VMHC of specific brain regions with significant differences were extracted and then correlated with one another. We also examined whether significant associations exist between any two of the metrics including rs-fMRI indices, clinical parameters and cognitive performance by calculating partial correlation coefficients using age, sex and education as covariates. Thresholds of correlation analysis were also set at *P* < 0.05, corrected by the Bonferroni’s method (the outcome acquired by dividing 0.05 by the number of statistical tests was the corrected threshold for each statistical test).

## Results

### Demographic characteristics

A total of 68 participants (34 patients and 34 control subjects) were finally enrolled for further statistical analysis (Table 1). All patients were regularly treated with oral antidiabetic agents. The two groups showed no significant difference in terms of age, sex, education, BMI, blood pressure, cholesterol or triglyceride level. Patients with T2DM had significantly higher HbAlc and fasting glucose levels. Control subjects showed a significantly lower level of low-density lipoprotein. Both groups showed similar performance in MoCA test and were all in the normal range (≥26).

**TABLE 1:** Demographic and clinical characteristics of subjects in patient and control groups.

Characteristics	Control subjects	T2DM patients	*P*-value
Age (years)	52.35 ± 6.76	52.44 ± 8.56	0.96
Sex (male/female)	12/22	17/17	0.22
Education (years)	9.24 ± 0.73	8.67 ± 1.24	0.15
Height (m)	1.65 ± 0.07	1.70 ± 0.06	0.07
Weight (kg)	69.85 ± 10.80	70.38 ± 9.68	0.83
BMI (kg/m^2^)	25.42 ± 2.90	24.37 ± 2.51	0.12
Disease duration (years)		8.18 ± 5.89	
MoCA	26.85 ± 0.96	26.64 ± 0.69	0.313
HbAlc (%)	5.05 ± 0.16	7.79 ± 1.59	<0.001
Fasting glucose (mmol/L)	5.49 ± 0.20	9.01 ± 2.48	<0.001
Cholesterol (mmol/L)	4.38 ± 0.76	4.13 ± 1.05	0.28
Triglyceride (mmol/L)	1.93 ± 1.26	1.46 ± 2.97	0.33
High density lipoprotein (mmol/L)	1.22 ± 0.36	1.14 ± 0.43	0.43
Low density lipoprotein (mmol/L)	2.76 ± 0.60	2.19 ± 0.89	0.57
Systolic blood pressure (mmHg)	119.47 ± 5.46	121.08 ± 6.29	0.26
Diastolic blood pressure (mmHg)	78.7 ± 5.1	77.4 ± 4.4	0.50

Data are presented as mean ± SD.

### Structural results

The two groups did not differ in GM, WM or brain parenchyma volumes. The VBM analysis didn’t indicate a significant difference in regional brain volume.

### ALFF, ReHo, and VMHC analyses

**FIGURE 1:**
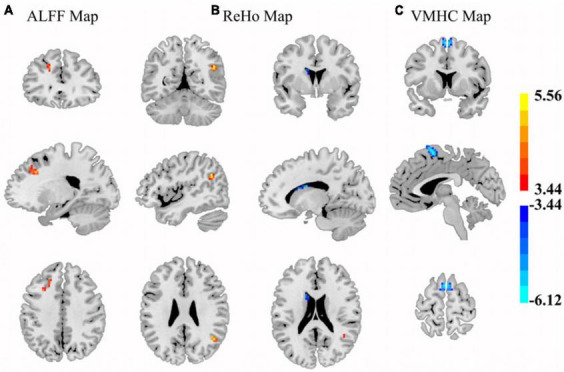
The brain regions where ALFF **(A)**, ReHo **(B)**, and VMHC **(C)** were significantly different by comparing the control subjects and T2DM patients (GRF corrected). **(A)** Control subjects exhibited higher ALFF values in left middle/superior frontal gyrus areas. **(B)** Control subjects showed increased ReHo values in right angular and decreased values in the left caudate. **(C)** Control subjects presented lower VMHC in the bilateral SMA. Color scales denote the *t*-value.

In T2DM patients, the ALFF values were significantly decreased in the left prefrontal cortex, mainly including middle frontal gyrus (MFG) and superior frontal gyrus (SFG). The ReHo values were also significantly decreased in the right angular gyrus. Higher ReHo and VMHC values were observed in left caudate and supplementary motor area (SMA), respectively (Figure 1 and Table 2).

**TABLE 2:** Differences in standard ALFF, ReHo, and VMHC values between the patient and control groups (*p* < 0.05, GRF corrected).

No. of voxel cluster	Brain regions	BA	Peak MNI (mm)			Peak *t*-value	Cluster size (voxels)
			**X**	**Y**	**Z**		
	**ALFF**						
1	L MFG/SFG	8/9	−21	24	36	4.652	38
	**ReHo**						
2	R Angular	39	24	−33	−45	5.556	18
3	L Caudate	–	−21	−39	−54	−6.122	15
	**VMHC**						
4	L SMA	6	−3	99	66	−4.734	46
5	R SMA	6	3	99	66	−4.734	46

L, left; R, right; MFG, middle frontal gyrus; SFG, superior frontal gyrus; SMA, Supplement motor area; BA, Brodmann area; MNI, Montreal Neurological Institute. Negative *t*-values: patients with T2DM > control subjects; Positive *t*-values: patients with T2DM < control subjects.

### Correlation analysis

In T2DM patients, no correlation between any two of the metrics including rs-fMRI indices, clinical parameters and cognitive performance survived the Bonferroni’s correction.

**FIGURE 2:**
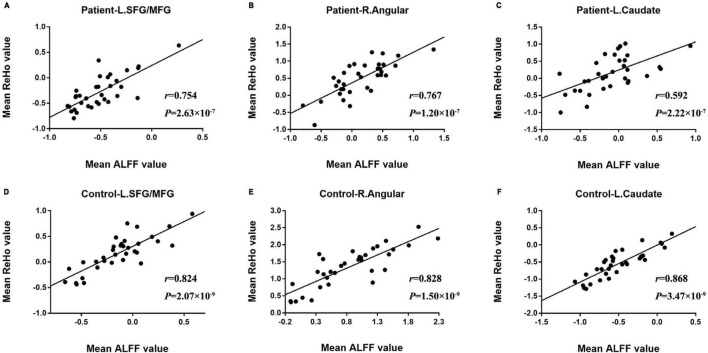
The comparison of significant correlations among mean values of rs-fMRI indices survived after Bonferroni’s correction in patients and control subjects. **(A–C)** Was the survived correlation between ALFF and ReHo values in L. SFG/MFG (left superior/middle frontal gyrus), R. Angular (right angular gyrus) and L. Caudate (left caudate), respectively. **(D–F)** Was the survived correlation observed in control subjects.

Significant correlations between rs-fMRI indices in the abnormal brain regions which survived Bonferroni’s correction were presented in Figure 2. The ALFF and ReHo values were significantly positively correlated in the in left prefrontal cortex, right angular gyrus and caudate. Meanwhile, a similar correlation relationship was observed in control group as well, but stronger than the patients group.

## Discussion

Our study demonstrated abnormal neural activity in specific brain regions in T2DM patients compared to control subjects. Neural abnormalities were primarily demonstrated in the occipital and prefrontal lobe, and decreased correlations between ALFF and ReHo indices were observed in these brain regions in patients.

Amplitude of low-frequency fluctuation, ReHo and VMHC analyses were based on different mechanisms. The ALFF analysis demonstrate the intensity of spontaneous neural activity within certain frequency range, namely 0.01∼0.08 Hz, where very-low-frequency drift and high frequency noise are removed (Yang et al., 2007). ReHo depicts the functional synchronization of neural activity in neighbor voxels (Zang et al., 2004). VMHC estimates the FC between homotopic voxels in two hemispheres (Zuo et al., 2010). Each method contributed partly elucidating the functional abnormalities in T2DM patients. In line with previous research, our findings show that a combined usage of ALFF/ReHo/VMHC is rational and necessary (Zhang et al., 2022). The absence of coexistence might be attributed to the intact cognitive function of the study cohort.

Significantly decreased ALFF values in the MFG/SFG were one of the major findings in our study. The MFG/SFG is generally considered a vital region mainly responsible for high-order cognitive function (Pizzagalli and Roberts, 2022). Human neuroimaging studies have demonstrated the MFG plays a critical role in the reorienting of attention, working memory and language comprehension (Briggs et al., 2021). And SFG is known to be involved in several functional networks related to motor activity, resting-state regulation and cognitive control (Briggs et al., 2020). In line with previous studies, the hypoactivation of neurons in the MFG/SFG area suggests a vulnerable region for T2DM (van Bussel et al., 2016; Xia et al., 2020). However, while decreased functional coherence and dynamic neural activity of MFG were found in diabetic brains (Guo et al., 2020; Xu et al., 2020), enhanced neural activities of SFG were observed in T2DM patients with diabetic retinopathy (Kim et al., 2016; Min et al., 2018; Shi et al., 2020). Therefore, both MFG/SFG were involved in the pathological process at very early stage, and the SFG was more susceptible to diabetic complications.

Decreased ReHo values in the angular gyrus were another notably finding in our study. The angular gyrus is a cross-modal integration hub involved in a variety of cognitive processes and mainly characterized by semantic processing (Seghier, 2023). Studies have confirmed that damage to the angular gyrus could produce a variety of cognitive dysfunctions (Qi et al., 2021). Reduced functional connectivity anchoring in the angular gyrus has been demonstrated in T2DM patients (Liu et al., 2018, 2020). Besides, patients with mild cognitive impairment could benefit from repetitive transcranial magnetic stimulation to the angular gyrus (Yang et al., 2022). Therefore, there is a good chance that the angular might serve as a very early neuroimaging biomarker for T2DM-related cognitive impairment.

Increased ReHo and VMHC values were observed in caudate and SMA, respectively, in T2DM patients. The caudate acts one of the hubs in frontostriatal circuits that have been implicated in behavior (Ji et al., 2018). Previous study indicated that deficits in prefrontal-striatal sensorimotor loops involving the caudate nucleus might be the neuropathological basis of motor and sensory dysfunction in T2DM (Fu et al., 2022). And these decreased connectivities might underlie certain abnormalities that occur in cognitive and emotional disorders (Barber et al., 2019). The inconsistent hyperactivity might be attributed to the compensatory mechanisms often appearing in the prediabetes and the early stage T2DM before cognitive impairment (Liu et al., 2019). The same goes for the SMA, which is mainly associated with motor programming. Meta-analysis showed increased cerebral blood flow in the SMA in T2DM (Liu J. et al., 2022), further supporting a working compensation mechanism in the diabetic brains.

Reduced correlation strength between average ALFF and ReHo values extracted from left MTG/SFG, caudate and right angular gyrus were observed in T2DM patients when compared with control subjects. This reduction of functional concordance, or “decoupling,” could be a manifestation of ongoing pathological changes underlying cognitive impairment. The decoupling gave extra evidences demonstrating that these brain regions were more sensitive to diabetes and might play a critical role in diabetes-related cognitive dysfunction at early stage. Except for the correlations between rs-fMRI indices, no significant correlation between rs-fMRI indices and clinical parameters or cognitive performance was observed. Studies focusing on T2DM patients without cognitive impairment detected little direct relationship between cognitive performances and rs-fMRI measurements (Liu et al., 2017, 2018; Kang et al., 2022). A task-fMRI based study indicated mediation effects among olfactory dysfunction, pancreatic function and executive function in T2DM patients without cognitive impairment (Zhang et al., 2018). This is a reminder that more correlation could be revealed by methodologies beyond rs-fMRI studies.

Insulin has been increasing recognized to be involved in the cognitive processes such as attention, executive functioning, learning and memory (Cui et al., 2022a). A number of studies have investigated the relationship between insulin resistance, brain MRI and cognitive function. Decreased sensitivity to insulin action is the major feature of T2DM and insulin resistance has also been observed in patients with Alzheimer’s disease. In previous studies, the HOMA-IR index was negatively correlated with spontaneous neural activity in specific brain regions (Xia et al., 2013; Cui et al., 2014; Zhang et al., 2019). In this study we mainly enrolled T2DM subjects from outpatients, missing the IR tests. In the next step we will include more hospitalized patients and supplement the IR data to investigate the relationship between IR and rs-fMRI indices in diabetic brains at early stage.

This study has several limitations. Firstly, the T2DM patients were receiving various anti-diabetic drugs, which may affect the neural activity (Cukierman-Yaffe et al., 2020; Verhulst et al., 2022). We hope to enroll drug-naïve patients initially diagnosed with T2DM to rule out the bias of medication in further studies. Secondly, the sample size of this study was small. This may be the one of the reasons that differences exist in results compared to former studies. Further studies should include more participants to verify the robustness of our results and detect more neural activity changes. Thirdly, our study didn’t carry more detailed tests to examine the sub-function of cognitive function. More information about cognition changes may be helpful to investigate the undiscovered correlation between brain changes and cognitive function.

## Conclusion

In conclusion, the combination of ALFF, ReHo, and VMHC analyses demonstrated abnormal spontaneous neural activity in different brain regions, respectively. Decreased neural activities were mainly observed in right angular gyrus and left prefrontal cortex while increased neural activities were mainly found in the SMA and left caudate. Furthermore, a decreased correlation between ALFF and ReHo was also observed in T2DM patients compared to control subjects. These results enhance our understanding of the diabetic brain changes that prior to cognitive impairment.

## Data availability statement

The raw data supporting the conclusions of this article will be made available by the authors, without undue reservation.

## Ethics statement

The studies involving humans were approved by the Ethics Committee of Beijing YouAn Hospital affiliated to Capital Medical University. The studies were conducted in accordance with the local legislation and institutional requirements. The participants provided their written informed consent to participate in this study.

## Author contributions

QW: Investigation, Writing – original draft, Writing – review and editing. CH: Formal analysis, Methodology, Writing – original draft, Writing – review and editing. XJ: Data curation, Validation, Writing – original draft. HL: Funding acquisition, Investigation, Resources, Supervision, Writing – original draft, Writing – review and editing.

## Footnotes

1
https://www.fil.ion.ucl.ac.uk/spm/software/spm12/
